# How to increase awareness of additional vaccinations; the case of maternal pertussis vaccination

**DOI:** 10.1186/s12889-021-11344-0

**Published:** 2021-06-29

**Authors:** K. van Zoonen, W. L. M. Ruijs, H. E. De Melker, M. E. J. Bongers, L. Mollema

**Affiliations:** Centre for Infectious Disease Control, National Institute for Public Health and the Environment (RIVM), PO Box 1, 3720 BA Bilthoven, The Netherlands

## Abstract

**Background:**

Effective and safe vaccines are available outside national immunization programs (NIP). Increased awareness and vaccine uptake can improve public health. Before the inclusion of maternal pertussis vaccination (MPV) in the Dutch NIP in December 2019, extra communication efforts were undertaken. Here we examine the success of these efforts, investigating women’s awareness of and their decision-making process regarding MPV.

**Methods:**

Between December 2018 and January 2019, one year before the introduction of MPV in the NIP, and about three years after MPV was recommended by the Dutch Health Council, pregnant and non-pregnant women (i.e. child younger than two years) were invited to fill out an online questionnaire. Participant’s decision-making processes regarding MPV were assessed with an adapted Precaution Adoption Process Model (PAPM), including stages of awareness, engagement, information-seeking, and vaccination behaviour. Furthermore, factors related to the decision-making process were examined.

**Results:**

In total, 942 women were included, of whom 62% were non-pregnant. Most of the pregnant and nonpregnant women were aware of MPV during pregnancy (respectively 69 and 56%). Most aware women had heard about MPV through their midwife and the Public Health Institute (PHI) website. Women unaware of MPV reported a need for information, preferably from their midwives. Most aware women felt MPV was important to them (88%) and were classified as “engaged”. Of the eligible and “engaged” pregnant women, 58% were vaccinated, versus 38% of “engaged” non-pregnant women.

**Conclusions:**

As the most preferred and trusted source of information, midwives are essential to increasing awareness of MPV. The PHI website is considered to be a reliable information source and is often consulted. To increase awareness, appropriate healthcare workers should be encouraged to actively inform target groups about available, additional vaccinations.

**Supplementary Information:**

The online version contains supplementary material available at 10.1186/s12889-021-11344-0.

## Background

Comprehensive routine vaccination programmes are in place in many countries worldwide [1]. However, there are additional, effective, and safe vaccines available that are not yet recommended or included in these programmes. In the Netherlands, the National Immunization Program (NIP) offers vaccines free of charge, and on a voluntary basis. Additional vaccines outside of the NIP are available to the public through out-of-pocket purchases. Information regarding these additional vaccines is provided on the website of the Dutch public health institute (PHI). Additional vaccines are not actively promoted, and consequently both public and health professional awareness is low. With increased awareness, and subsequently increased uptake, of vaccines outside of the NIP there are improvements in public health to be gained [2].

Awareness, information seeking, and engagement are essential to the vaccination decision-making process. The process can be explained within the framework of the Precaution Adoption Process Model (PAPM). The model demonstrates how risk mediation mechanisms form pathways to health-protective behaviours, such as vaccination [3, 4]. The PAPM consists of seven stages from “unaware” to “action” to “maintenance” of health-protective behaviours. The model also identifies factors (such as barriers to health care, self-efficacy, trust in information sources, decision-making styles, educational level, attitude, and intention) that facilitate or impede movement through the seven stages [3, 5–7].

Since 2015 the Dutch Health Council has recommended a Diphtheria, Tetanus, and acellular Pertussis (dTaP) vaccine for pregnant women, available as an out-of-pocket purchase. In December 2019, the maternal pertussis vaccination (MPV) was included as part of the NIP, for administration between 28 to 36 weeks of pregnancy [8]. Between 2015 and 2019, there were different routes for pregnant women to obtain the NIP provided MPV in the Netherlands. In some regions, women had to consult with their general practitioner (GP) to obtain a prescription for the MPV for collection at the local pharmacy. In other regions, women could contact municipal public health services (PHS) directly to receive their vaccine. If a mother is vaccinated during pregnancy her infant would follow a 3, 5, 11-month vaccination schedule, as opposed to the 2, 3, 5, 11-month vaccination schedule for infants of unvaccinated mothers (see: Dutch National Immunisation Programme | Rijksvaccinatieprogramma.nl).

In 2017, prompted by the media attention surrounding the MPV, and following the advice of the Dutch Health Council, the Dutch PHI decided to undertake supplementary communication efforts to increase MPV awareness. Communication efforts were directed at pregnant women and their antenatal care providers. In the Netherlands antenatal care is the responsibility of midwives and/or gynaecologists, and not GPs. Furthermore, GPs are not responsible for administering NIP vaccinations, which are instead delivered by youth health workers (YHW). As such, from 2017 flyers and letters containing MPV information were delivered directly to pregnant women by midwives and gynaecologists. In 2018 further flyers, factsheets and posters were distributed amongst professionals as well as for display in the waiting rooms of primary care services and youth health worker’s offices (i.e. PHS).

In this study, we firstly aim to examine the awareness of women regarding MPV, to investigate the success of the 2017/2018 MPV communication efforts. Secondly, we analyse the decision-making process of pregnant women concerning the uptake of the MPV. We aim to identify potential demographic and socio-cognitive factors that may mediate each stage of the MPV uptake decision-making process, within the framework of the PAPM. The results of this study can contribute to improving national, and international, communication efforts to increase awareness and vaccine uptake of non-NIP vaccines.

## Methods

### Study population and recruitment

Between December 2018 and January 2019, women between 18 to 49 years of age were invited to fill out an online survey (see supplementary materials). Invitees were women who were pregnant at the time of the study and women who had been recently pregnant. Recent pregnancy was defined based on the age of the youngest child and was limited to 2 years of age to minimise recall bias. This age limit was also chosen as active MPV-related information materials (e.g. website, fora, and magazines focusing on pregnant women) were available from 2017 onwards – two years before the study. Additionally, the distribution of information flyers and factsheets at GPs and YHWs offices began in 2018. Women were directly recruited through an online survey panel (Flycatcher, ISO 26362, https://www.flycatcherpanel.nl/nld/over-het-panel/). Participants also recruited other women not included in the original sampling frame, a method known as snowball sampling. All participants provided active consent irrespective of their recruitment route. All survey questions related to the current, or the most recent, pregnancy of the participant.

This study was not subject to the law in the Netherlands regarding medical research involving human subjects according to The Clinical Expertise Centre RIVM (No. LCI-423). Therefore, this study was exempt from requiring further approval from an ethics research committee.

### The precaution adaptation model (PAPM)

**Fig. 1:**
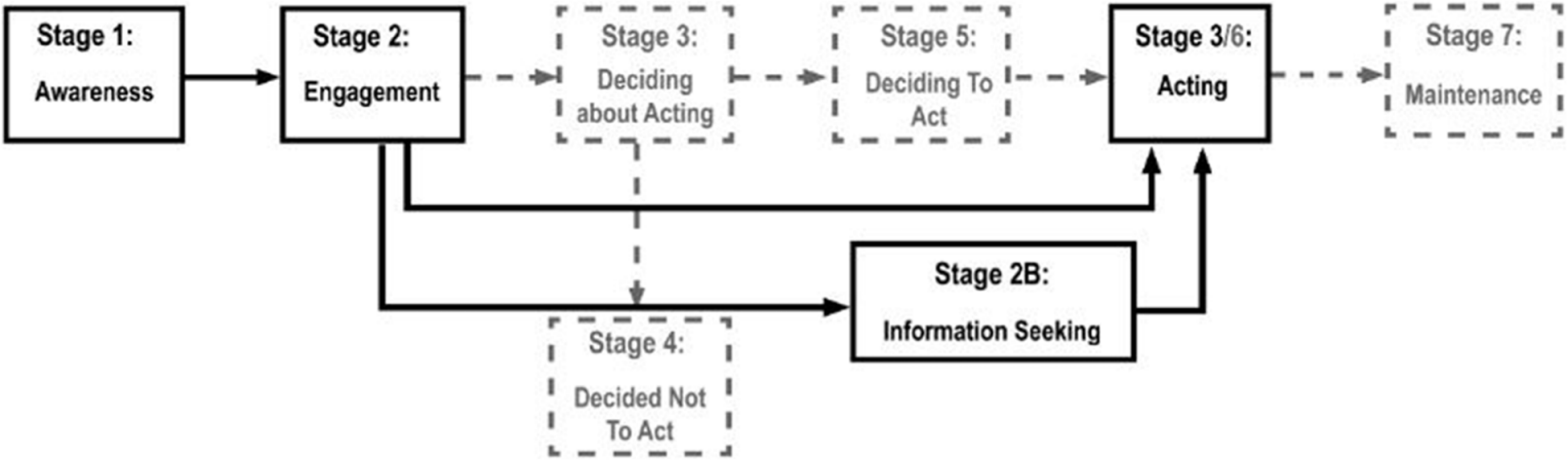
Original complete PAPM (black and greyscale boxes and arrows) and the model adapted from the PAPM for this study (black boxes and arrows only)

The PAPM was used as a theoretical framework to develop the survey. We adapted the model to investigate two behaviours pertinent to MPV awareness and uptake: information seeking and vaccination behaviour (Fig. 1). Stage 1 of the adapted model examines the awareness of the MPV. In Stage 2 women engage with the decision-making process of whether or not the MPV is important or relevant to them. Stage 2 can only be entered once women become aware of the MPV, in other words, once women have entered Stage 1 [3]. After Stage 2, women can enter Stage 2B and search for (additional) information to support the decision-making process, and subsequently enter Stage 3, at which point the decision to vaccinate or not is made. Women can also bypass Stage 2B and enter Stage 3 directly from Stage 2 (Fig. 1). Stage 2B and Stage 3 can only be entered after women have engaged with the MPV in Stage 2. Stages 2B and 3 of the adapted model replaced Stage 3 through to 6 of the original model and sage 7 of the original PAPM was excluded as vaccination with the MPV is not a recurrent or maintained behaviour (Fig. 1).

### Survey

A pilot survey was conducted to optimize the final questionnaire and ensure the questions and answers were comprehensive and logical. Questions regarding demographics (e.g. age, education, ethnicity), awareness of the MPV, information delivery preferences (i.e. if women were unaware, would they have preferred to hear or read about MPV), engagement with the MPV, MPV information-seeking behaviour, and vaccination uptake were included in the final questionnaire. In addition, the survey included questions regarding factors that may influence the individual stages of the adapted PAPM. For Stage 1 questions examined MPV information sources, formats, and individual preference for information format. In Stage 2, Stage 2B, and Stage 3 questions assessed participant critical thinking (i.e. the ability to distinguish different information about MPV and take into account the reliability of an information source), descriptive norm (i.e. most women will get vaccinated during their pregnancy) and participant trust in three information sources (midwives, PHI and PHS). Stage 2b and Stage 3 included further questions regarding participant self-efficacy, injunctive norms, considerations (i.e. possible barriers or facilitators). For Stage 2B the duration of search for information, information sources, trust in information sources, and subjective knowledge of the MPV was examined. For Stage 3, attitudes, intentions, information-seeking behaviours, and vaccine provider were examined. This section of the survey also explored reasons for deciding about MPV. This was done using 6 subjective consideration statements. Three subgroups of statements investigated reasons not to vaccinate. The first group of statements related to possible advantages for the participant’s baby, and included the statements “If my baby would need one vaccination less, I would get MPV” and “If the baby would get its first vaccinations later in life, I would get MPV”. The second group of statements considered the route of MPV administration, and included the statements “I would get the vaccination if it is provided by my midwife”, “If MPV is provided by someone other than my GP or midwife I would not go”, and “If MPV is included in the NIP I would get it”. The final group of statements related to the accessibility for obtaining MPV, and included the statement “If I wanted to get the MPV I would know what to do”.

For both Stage 2B and Stage 3 decision-making styles were investigated [9, 10]. To identify the decision-making styles of the participants the Dutch version of the General Decision Making Style (GDMS) was included in the survey. It consists of 25 items corresponding to five decision making styles; i) a rational style with emphasis on a thorough and logical process, ii) intuitive style with emphasis on intuition and inner feelings, iii) dependent style with emphasis on relying on the advice of others, iv) avoidant style with emphasis on avoiding making a decision, and v) impulsive (or spontaneous) style with emphasis on making a decision as soon as possible [11, 12]. These styles are independent, but not mutually exclusive [13].

### Statistical analyses

Firstly, for each survey item, the Cronbach’s alpha (α) was identified, and scales were adjusted to ensure each survey item was summed to one scale (see Appendix Table 1). Survey items relating to injunctive norms were analysed separately due to their relatively low Cronbach’s alpha (0.66). Within the subjective considerations question panel the survey item “If MPV is provided by someone other than my GP or midwife I would not go” was recoded to match the scale of the other subjective consideration statements.

Secondly, MPV information materials (e.g. website, fora, and magazines focusing on pregnant women) became available in 2017, and additional information flyers and factsheets were distributed from 2018. As such, non-pregnant women probably had less access to certain relevant information materials. Therefore, analyses per PAPM stage were conducted separately for pregnant and non-pregnant women. All data analyses were performed in SPSS for Windows (version 24.0). Chi-squared tests, independent sample t-tests, and ANOVA’s were conducted. If cell counts were low, the categories were recoded, and the Welch test was examined when Levene’s test was significant.

## Results

### Overall

In total, 947 women completed the questionnaire. 5 women were excluded as they fell outside of the age range (18–49 years) [14]. Therefore, the net survey sample was 942 women (Table 1).

**Table 1:** Overall descriptive statistics

Variable	Total Sample N = 942 (100%)	Pregnant ***N*** = 358 (38%)	Non-pregnant ***N*** = 584 (62%)
Age (mean, SD)	31.8 (4.7)	30.8 (5.4)	32.4 (4.2)**
Ethnicity (n, %)			
Dutch	884 (93.8)	332 (92.7)	552 (94.5)
Other	58 (6.2)	26 (7.3)	32 (5.5)
Education (n, %)			
Low	83 (8.8)	37 (10.3)	46 (7.9)
Middle	228 (24.2)	97 (27.1)	131 (22.4)
High	631 (67.0)	224 (62.6)	407 (69.7)
One or more children (n, %)			
Yes	797 (84.6)	213 (59.5)	584 (100)
No	145 (15.4)	145 (40.5)	N/A
Age youngest child (n, %)			
0–2 years	646 (81.1)	62 (29.1)	584 (100)
2–4 years	98 (12.3)	98 (46.0)	N/A
4–8 years	35 (4.4)	35 (16.4)	N/A
8–12 years	13 (1.6)	13 (6.1)	N/A
12–18 years	3 (0.4)	3 (1.4)	N/A
18+ years	2 (0.3)	2 (0.9)	N/A
Occupation as Health care worker (n, %)			
Yes	340 (36.1)	125 (34.9)	215 (36.8)
No	602 (63.9)	233 (65.1)	369 (63.2)
Awareness of MPV (n, %)			
Aware	576 (61,1)	248 (69,3)	328 (56,2)**
Unaware	366 (38,9)	110 (30,7)	256 (43,8)**
Engagement with MPV (n, %)			
Unengaged	147 (15,6)	49 (13,7)	98 (16,8)
Neutral	240 (25,5)	83 (23,2)	157 (26,9)
Engaged	555 (58,9)	226 (63,1)	329 (56,3)
Information seeking (n, %)			
Yes	347 (36,8)	153 (44,7)	194 (36,8)*
No	522 (55,4)	189 (55,3)	333 (63,2)*
Missing	73 (7,7)	16 (N/A)	57 (N/A)
Vaccinated (n, %)			
Yes	227 (24,1)	111 (31)	116 (19,9)**
No	715 (75,9)	247 (69)	468 (80,1)**

c N/A = not applicable. * < .05; ** *p* < .000

The average age of participants was 31.8 years. The majority of participants were highly educated (67%) and most were of Dutch nationality (94%). Most women completed the questionnaire through Flycatcher (84%). The majority of women were non-pregnant (62%). Non-pregnant women were on average older than pregnant women (32.4 years vs 30.8 years respectively). Of the 358 pregnant women, nearly 60% already had an older child. The average gestational age of pregnant participants was 21 weeks (range 2–42 weeks). When comparing non-pregnant women to pregnant women, pregnant women were more frequently aware of the MPV (69.3% vs 56.2%, *N* = 942), sought information about the MPV (44.7% vs 36.8%, N = 942) and were more often vaccinated with the MPV (31% vs 19.9%, N = 942). The percentages of neutral and engaged pregnant women were similar to the percentages of neutral and engaged non-pregnant women (86.3% vs 83.2% respectively, N = 942).

#### Stage 1: awareness of MPV

Approximately 69% of pregnant women and 56% of non-pregnant women were aware of MPV (Table 2). Of those, most women had heard about MPV through their midwife (49% of pregnant women and 39% of non-pregnant women). However, pregnant women also reported that other health care workers (HCW), such as GPs or PHS, had been their MPV information sources. Non-pregnant women reported the PHI website was an important information source. Non-pregnant women reported they would prefer to be informed about the MPV through the internet (35%), whereas pregnant women reported preferences for information delivery through the internet (35%) or in consultation with a HCW (35%). Pregnant women who already had a child, and non-pregnant women who were HCW themselves were more likely to be aware of the MPV (64 and 42% respectively).

**Table 2:** Descriptive statistics Stage 1: Awareness of MPV (overall aware *N* = 576)

	Pregnant (N = 358)		Non-pregnant (N = 584)	
	Aware (*N* = 248 (69.3))	Unaware (*N* = 110 (30.7))	Aware (*N* = 328 (56.2))	Unaware (*N* = 256 (43.8))
Age (M, Sd)	31.1 (5.6)	30.3 (4.6)	32.2 (4.1)	32.6 (4.3)
Ethnicity (N, %)				
Dutch	227 (91.5)	105 (95.5)	309 (94.2)	243 (94.9)
Other	21 (8.5)	5 (4.5)	19 (5.8)	13 (5.1)
Education (N, %)				
Low	27 (10.9)	10 (9.1)	31 (9.5)	15 (5.9)
Middle	71 (28.6)	26 (23.6)	65 (19.8)	66 (25.8)
High	150 (60.5)	74 (67.3)	232 (70.7)	175 (68.4)
Occupation as a HCW (N, %)				
Yes	91 (36.7)	34 (30.9)	139 (42.4)	76 (29.7)**
No	157 (63.3)	76 (69.1)	189 (57.6)	180 (70.3)**
One or more children (N, %)				
Yes	159 (64.1)	54 (49.1)**	328 (100)	256 (100)
No	89 (35.9)	56 (50. 9)	N/A	N/A
Information source (N, %)		N/A		N/A
Midwife	121 (48.8)		129 (39.3)	
GP	36 (14.5)		28 (8.5)	
Municipal public health services	32 (12.9)		17 (5.2)	
Gynaecologist	18 (7.3)		28 (8.5)	
Youth health care worker	22 (8.9)		12 (3.7)	
Public Health Institute	22 (8.9)		46 (14.0)	
Government	22 (8.9)		23 (7.0)	
Pregnant women	45 (18.1)		69 (21.0)	
Other	49 (19.8)		91 (27.7)	
Information type (N, %)		N/A		N/A
Consultation HCW	86 (34.7)		99 (30.2)	
Information flyer about MPV	75 (30.2)		78 (23.8)	
Website of Public Health Institute	40 (16.1)		66 (20.1)	
Internet	86 (34.7)		114 (34.8)	
Social media	27 (10.9)		41 (12.5)	
Traditional media	18 (7.3)		45 (13.7)	
Centering pregnancy	20 (8.1)		31 (9.5)	
Other	24 (9.7)		34 (10.4)	
Information need (N, %)	N/A		N/A	
Yes		94 (85.5)		199 (77.7)
No		16 (14.5)		57 (22.3)
Information source (N, %)^*a*^	N/A		N/A	
Midwife		65 (69.1)		150 (75.4)
GP		11 (11.7)		3 (1.5)
Municipal public health services		3 (3.2)		3 (1.5)
Gynaecologist		9 (9.6)		26 (13.1)
Youth health care worker		1 (1.1)		6 (3.0)
Public Health Institute		2 (2.1)		8 (4.0)
Government		–		3 (1.5)
Pregnant women		2 (2.1)		–
Other		1 (1.1)		–
Information type (N, %)^*a*^	N/A		N/A	
Consultation HCW		38 (40.4)		88 (44.2)
Information flyer about MPV		38 (40.4)		86 (43.2)
Additional overall information flyer		10 (10.6)		21 (10.6)
Website of Public Health Institute		3 (3.2)		1 (0.5)
Through e-mail		3 (3.2)		2 (1.0)
Through Mobile app		1 (1.1)		–
Government campaign		–		1 (0.5)
Other		1 (1.1)		–

^a^ routing only when need for information was yes. M = Mean; Sd = standard deviation; N = number of participants; % = percentage of participants; N/A = not applicable. * *p* < .05; ** *p* < .01

Examining the women who were unaware of MPV, the majority of both pregnant and non-pregnant women reported a need for information and preferred the midwife to be the source of MPV information. For non-pregnant women, the second most preferred information source after midwives was their gynaecologist (13%), and for pregnant women their GP (12%). Furthermore, both pregnant and non-pregnant preferred to receive information through a consultation with a HCW or to receive a flyer about the MPV. Women who reported to be unaware of the MPV, and felt no need to be informed, reported mostly that they were not interested or felt the MPV was unnecessary (respectively 37.5 and 22.8%). Pregnant women who were not aware of the MPV also reported having received too much information regarding all aspects of pregnancy overall and felt that the MPV was not relevant to them at the current point of their pregnancy.

#### Stage 2: engagement with MPV

Nearly a third of both pregnant and non-pregnant women felt MPV was not important to them and were thus considered “unengaged” (Table 3). The women who were engaged with MPV were significantly older than the unengaged group. Engaged women also had more trust in their midwives, PHI and PHS. Furthermore, women engaged with MPV scored higher in measures of critical thinking (i.e. ability to distinguish different information about MPV and take into account the reliability of the information source) and descriptive norms (i.e. felt that most women will get vaccinated during their pregnancy) compared to women who were neutral or unengaged.

**Table 3:** Descriptive statistics stage 2: Engagement with MPV (Overall engaged *N* = 500)

	Pregnant (N = 248)			Non-pregnant (N = 328)		
	Unengaged (*N* = 31 (12.5))	Neutral (N = 50 (20.2))	Engaged (*N* = 167 (67.3))	Unengaged (*N* = 45 (13.7))	Neutral (*N* = 74 (22.6))	Engaged (*N* = 209 (63.7))
Age (M, Sd)	30.1 (4.2)	29.5 (5.4)^e^	31.7 (5.8)*^n^	30.7 (3.4)^e^	31.7 (3.8)	32.7 (4.3)**^u^
Ethnicity (N, %)						
Dutch	28 (90.3)	45 (90.0)	154 (92.2)	42 (93.3)	70 (94.6)	197 (94.3)
Other	3 (9.7)	5 (10.0)	13 (7.8)	3 (6.7)	4 (5.4)	12 (5.7)
Education (N, %)						
Low & middle	11 (35.5)	23 (46)	64 (38.3)	18 (40.0)	23 (31.1)	55 (26.3)
High	20 (64.5)	27 (54)	103 (61.7)	27 (60.0)	51 (68.9)	154 (73.7)
Occupation as a HCW (N, %)						
Yes	12 (38.7)	13 (26.0)	66 (39.5)	19 (42.2)	28 (37.8)	92 (44.0)
No	19 (61.3)	37 (74.0)	101 (60.5)	26 (57.8)	46 (62.2)	117 (56.0)
One or more children (N, %)						
Yes	19 (61.3)	25 (50.0)^e^	115 (68.9)*^n^	45 (100)	74 (100)	209 (100)
No	12 (38.7)	25 (50.0)^e^	52 (31.1)*^n^	N/A	N/A	N/A
Critical thinking^a^ (M, Sd)	4.8 (1.5)^e^	4.8 (1.0)^e^	5.5 (0.9)***^u.n^	4.9 (1.1)^e^	5.1 (0.9)^e^	5.9 (0.8)***^u.n^
Descriptive norm^a^ (M, Sd)	3.7 (1.5)^e^	4.1 (1.0)^e^	4.9 (1.1)***^u.n^	3.9 (1.4)^e^	4.1 (1.2)^e^	4.8 (1.2)***^u.n^
Trust (M, Sd)						
Midwife	4.2 (1.5)^n.e^	5.1 (1.2)^u.e^	5.7 (1.1)***^u.n^	5.4 (1.2)	5.4 (1.1)^e^	5.8 (1.2)*^n^
Public Health Institute	3.7 (1.7)^n.e^	5.1 (1.2) ^u.e^	5.7 (0.9)***^u.n^	5.1 (1.6)^e^	5.4 (1.1)^e^	6.0 (1.1)***^u.n^
Youth health care centre	3.4 (1.8)^n.e^	4.8 (1.1) ^u.e^	5.3 (1.1)***^u.n^	4.7 (1.5)^e^	4.9 (1.2)^e^	5.4 (1.3)***^u.n^

M = Mean; Sd = standard deviation; N = number of participants; % = percentage of participants; u = unengaged; n = neutral; e = engaged; * p < .05; ** p < .01;*** *p* < .001. ^a^ scale 1–7

#### Stage 2b: information-seeking behaviour

Only women categorised as neutral or engaged in Stage 2 can enter Stage 2b of information-seeking behaviour. Of the neutral and engaged women, about 60% had actively searched for information regarding MPV (Table 4). Women who had searched for information were mostly higher educated and scored higher on self-efficacy (i.e. considered themselves able to decide MPV). Information-seeking women also scored higher in measures of critical thinking, subjective knowledge (i.e. how good they thought their knowledge about MPV was), and trust in the PHI. These women were most often classified as having a rational decision-making style.

**Table 4:** Descriptive statistics stage 2b: Information seeking behaviour (Overall *N* = 307 searched for information)

	Pregnant (***N*** = 217)		Non-pregnant (***N*** = 283)	
	Sought information (*N* = 131 (60.4))	Did not seek information (*N* = 86 (39.6))	Sought information (*N* = 176 (62.2))	Did not seek information (*N* = 107 (37.8))
Age (M, Sd)	31.2 (5.7)	31.3 (6.0)	32.6 (4.1)	32.3 (4.4)
Ethnicity (N, %)				
Dutch	120 (91.6)	79 (91.9)	171 (97.2)	96 (89.7)**
Other	11 (8.4)	7 (8.1)	5 (2.8)	11 (10.3)**
Education (N, %)				
Low	8 (6.1)	15 (17.4)**	11 (6.3)	17 (15.9)*
Middle	34 (26.0)	30 (34.9)	28 (15.9)	22 (20.6)
High	89 (67.9)	41 (47.7)**	137 (77.8)	68 (63.6)*
Occupation as a HCW (N, %)				
Yes	54 (41.2)	25 (29.1)	85 (48.3)	35 (32.7)*
No	77 (58.8)	61 (70.9)	91 (51.7)	72 (67.3)*
Children (N, %)				
Yes	84 (64.1)	56 (65.1)	176 (100)	107 (100)
No	47 (35.9)	30 (34.9)	N/A	N/A
Time of search (N, %)		N/A		N/A
Before pregnancy	17 (13.0)		6 (3.4)	
1st trimester	51 (38.9)		31 (17.6)	
2nd trimester	47 (35.9)		85 (48.3)	
3rd trimester	13 (9.9)		48 (27.3)	
After pregnancy	3 (2.3)		6 (3.4)	
Decision-making style (M, Sd)				
Rational	4.0 (0.5)	3.8 (0.6)**	4.1 (0.5)	3.9 (0.5)**
Intuitive	3.6 (0.7)	3.6 (0.7)	3.5 (0.7)	3.7 (0.7)
Dependent	3.6 (0.6)	3.6 (0.7)	3.4 (0.7)	3.4 (0.6)
Avoidant	2.8 (1.0)	2.8 (1.0)	2.2 (0.9)	2.5 (0.9)*
Impulsive	3.0 (0.8)	3.1 (0.7)	2.8 (0.7)	2.9 (0.7)
Critical Thinking^a^ (M, Sd)	5.6 (0.8)	4.9 (1.1)***	5.9 (0.8)	5.4 (0.9)***
Descriptive norm^a^ (M, Sd)	4.8 (1.1)	4.6 (1.2)	4.7 (1.2)	4.6 (1.2)
Injunctive norm^a^ (M, Sd)				
Loved ones	4.8 (1.3)	4.7 (1.4)	5.0 (1.2)	4.8 (1.2)
Health care worker	4.8 (1.4)	4.7 (1.4)	5.0 (1.3)	4.8 (1.2)
Trust (M, Sd)^a^				
Midwife	5.7 (1.1)	5.3 (1.2)*	5.7 (1.3)	5.6 (1.1)
Public Health Institute	5.7 (1.0)	5.2 (1.0)***	6.0 (1.1)	5.6 (1.1)**
Youth Health Care centre	5.3 (1.1)	5.1 (1.1)	5.3 (1.4)	5.3 (1.2)
Subjective Knowledge^a^ (M, Sd)	3.7 (0.7)	3.1 (1.0)***	3.8 (0.9)	3.0 (1.0)***
Self-efficacy (N, %)				
Yes	113 (86.3)	50 (58.1)***	142 (80.7)	65 (60.7)***
No	18 (13.7)	36 (41.9)***	34 (19.3)	42 (39.3)***
Information source (N, %)		N/A		N/A
GP	25 (11.5)		22 (7.8)	
Midwife/Gynaecologist	42 (19.4)		41 (14.5)	
Youth health care worker	12 (5.5)		9 (3.2)	
Municipal public health services	19 (8.8)		22 (7.8)	
Website of Public Health Institute	74 (34.1)		130 (45.9)	
Other Website	15 (6.9)		33 (11.7)	
Scientific articles	11 (5.1)		16 (5.7)	
Television	2 (0.9)		3 (1.1)	
Newspaper/magazine	2 (0.9)		1 (0.4)	
Pregnant women	18 (8.3)		18 (6.4)	
Social media	9 (4.1)		14 (4.9)	
Family/friends	10 (4.6)		6 (2.1)	
Other	4 (1.8)		2 (0.7)	
Information Trust^a^ (M, Sd)		N/A		N/A
GP	5.8 (1.0)		5.1 (1.8)	
Midwife/Gynaecologist	5.8 (1.1)		5.7 (1.3)	
Youth health care worker	6.0 (0.9)		5.4 (1.3)	
Municipal public health services	5.7 (1.0)		6.1 (0.9)	
Website of Public Health Institute	6.0 (0.8)		5.9 (1.4)	
Other Website	4.4 (0.5)		4.6 (1.3)	
Scientific articles	4.9 (1.8)		5.9 (1.3)	
Television	5.0 (0.0)		5.0 (1.0)	
Newspaper/magazine	5.0 (1.4)		3.0 (N/A)	
Pregnant women	4.5 (0.9)		4.1 (1.5)	
Social media	3.6 (0.7)		3.7 (1.5)	
Family/friends	5.9 (1.1)		5.8 (0.8)	
Other	5.8 (1.9)		4.5 (0.7)	

M = Mean; Sd = standard deviation; N = number of participants; % = percentage of participants; N/A = not applicable. * *p* < .05; ** *p* < .01; ***p < .0001. ^a^ scale 1–7

The PHI website was most often used as a source for (additional) information in both pregnant and non-pregnant women (34.1% vs 45.9% respectively), followed by their midwife/gynaecologist (19.4% vs 14.5% respectively). Furthermore, pregnant women reported that their GP was a source of MPV information (11.5%), while non-pregnant women used other websites (11.7%). These websites were mostly Google or forum-type websites. However, trust in “other websites” was lower compared to other information sources.

Non-pregnant women who had searched for information were more often of Dutch descent, and/or were in the health care profession. Non-pregnant women who did not search for information mostly had an avoidant decision-making style. Pregnant women who searched for information mostly did so during their first (38.9%) or second (35.9%) trimester and reported higher trust in their midwives.

#### Stage 3: vaccination behaviour (i.e. acting)

Only women with a neutral or engaged score in Stage 2 can enter this stage, irrespective of passing through Stage 2B. Of those, approximately 43% of pregnant women and 38% of non-pregnant women were vaccinated with the MPV during their most current or most recent pregnancy (Table 5). Of the currently pregnant women, only 33% were eligible for MPV based on their reported gestational age (28–36 weeks pregnant), of whom 58% were vaccinated.

**Table 5:** Descriptive statistics stage 3: Vaccine uptake (Overall *N* = 199 were vaccinated)

	Pregnant (N = 217)		Non-pregnant (N = 283)	
	Vaccinated (*N* = 93 (42.9))	Not vaccinated (*N* = 124 (57.1))	Vaccinated (*N* = 106 (37.5))	Not vaccinated (*N* = 177 (62.5))
Age (M, Sd)	31.7 (6.2)	30.9 (5.5)	32.6 (4.7)	32.4 (3.8)
Ethnicity (N, %)				
Dutch	82 (88.2)	117 (94.4)	96 (90.6)	171 (96.6)*
Other	11 (11.8)	7 (5.6)	10 (9.4)	6 (3.4)*
Education (N, %)				
Low	12 (12.9)	11 (8.9)	7 (6.6)	21 (11.9)
Middle	37 (39.8)	27 (21.8)**	15 (14.2)	35 (19.8)
High	44 (47.3)	86 (69.4)**	84 (79.2)	121 (68.4)
Occupation as a HCW (N, %)				
Yes	36 (38.7)	43 (34.7)	48 (45.3)	72 (40.7)
No	57 (61.3)	81 (65.3)	58 (54.7)	105 (59.3)
Children (N, %)				
Yes	67 (72.0)	73 (58.9)*	106 (100)	177 (100)
No	26 (28.0)	51 (41.1)*	N/A	N/A
Age youngest child (N, %)				
0–2 yrs.	16 (23.9)	22 (30.1)	106 (100)	177 (100)
2–4 yrs.	28 (41.8)	38 (52.1)	N/A	N/A
4–8 yrs.	11 (16.4)	11 (15.1)	N/A	N/A
8–12 yrs.	10 (14.9)	2 (2.7)	N/A	N/A
12–18 yrs.	2 (3.0)	0 (0)	N/A	N/A
18 yrs. and older	0 (0)	0 (0)	N/A	N/A
Considerations^a^ (M, Sd)				
1) Baby 1 less vaccination	5.5 (1.2)	5.1 (1.4)*	5.6 (1.6)	5.1 (1.6)*
2) Receive vaccine midwife	5.4 (1.1)	4.9 (1.6)**	5.6 (1.4)	5.1 (1.6)**
3) Know how to get MPV	5.6 (1.1)	4.9 (1.5)***	6.1 (1.1)	4.9 (1.6)***
4) MPV included in NIP	5.5 (1.3)	5.3 (1.6)	5.5 (1.5)	5.3 (1.5)
5) Baby vaccinated later age	5.4 (1.1)	5.0 (1.6)*	5.4 (1.7)	5.1 (1.7)
6) MPV not through youth health care centre	5.5 (1.3)	5.3 (1.2)	5.9 (1.2)	5.6 (1.2)
Decision-making style^a^ (M, Sd)				
Rational	4.0 (0.5)	3.9 (0.5)	4.1 (0.5)	4.0 (0.5)
Intuitive	3.7 (0.7)	3.6 (0.7)	3.6 (0.7)	3.6 (0.7)
Dependent	3.7 (0.6)	3.6 (0.7)	3.5 (0.7)	3.4 (0.6)
Avoidant	3.0 (1.0)	2.6 (0.9)**	2.3 (1.0)	2.4 (0.9)
Impulsive	3.2 (0.8)	2.9 (0.7)**	2.9 (0.7)	2.8 (0.7)
Critical Thinking^a^ (M, Sd)	5.5 (0.9)	5.2 (1.0)*	5.9 (0.9)	5.6 (0.9)**
Descriptive norm^a^ (M, Sd)	5.1 (1.0)	4.5 (1.1)***	5.0 (1.1)	4.5 (1.2)***
Injunctive norm^a^ (M, Sd)				
Loved ones	5.2 (1.1)	4.4 (1.3)***	5.4 (1.1)	4.7 (1.2)***
HCW	5.2 (1.3)	4.5 (1.4)**	5.3 (1.3)	4.7 (1.3)***
Trust^a^ (M, Sd)				
Midwife	5.8 (1.0)	5.4 (1.2)**	5.7 (1.3)	5.7 (1.1)
Public Health Institute	5.7 (1.0)	5.4 (1.1)*	6.0 (1.1)	5.8 (1.1)
Youth Health Care centre	5.4 (0.9)	5.0 (1.2)**	5.3 (1.4)	5.3 (1.3)
Subjective Knowledge^a^ (M, Sd)	3.9 (0.6)	3.2 (0.9)***	4.0 (0.8)	3.2 (1.0)***
Intention^a^ (M, Sd)	5.8 (1.1)	4.8 (1.5)***	6.3 (1.1)	4.9 (1.6)***
Attitude^a^ (M, Sd)	5.4 (1.0)	4.8 (1.1)***	5.9 (1.0)	5.0 (1.3)***
Self-efficacy (N, %)				
Yes	83 (89.2)	80 (64.5)***	101 (95.3)	106 (59.9)***
No	10 (10.8)	44 (35.5)***	5 (4.7)	71 (40.1)***
Information seeking (N, %)				
Yes	68 (73.1)	63 (50.8)**	78 (73.6)	98 (55.4)**
No	25 (26.9)	61 (49.2)**	28 (26.4)	79 (44.6)**
Vaccine provider (N, %)		N/A		N/A
GP	48 (51.6)		44 (41.5)	
Midwife	19 (20.4)		17 (16.0)	
Municipal public health services	22 (23.7)		35 (33.0)	
Other	4 (4.3)		10 (9.4)	
Reason for not vaccinating (N, %)	N/A		N/A	
Did not know MPV existed		36 (29.0)		34 (19.2)
Objections against MPV		9 (7.3)		12 (6.8)
Did not know enough about MPV		9 (7.3)		37 (20.9)
Couldn’t afford MPV		4 (3.2)		5 (2.8)
MPV is not included in NIP		19 (15.3)		34 (19.2)
MPV was not advised by HCW		11 (8.9)		29 (16.4)
Other		36 (29.0)		26 (14.7)

M = Mean; Sd = standard deviation; N = number of participants; % = percentage of participants; N/A = not applicable. * *p* < .05; ** *p* < .01; ***p < .0001. ^a^ scale 1–7

Of both pregnant and non-pregnant women, women who had searched for information and felt they knew enough to make a decision (i.e. self-efficacy) were more often vaccinated. Furthermore, both pregnant and non-pregnant vaccinated women more positive intention and attitude towards MPV felt better able to distinguish different information about MPV, expected other women to also get vaccinated during their pregnancies, and felt that loved ones and HCWs would appreciate it if they would get vaccinated during their pregnancy than non-vaccinated women. Vaccinated women also felt their knowledge about MPV was better, and compared to non-vaccinated women the following considerations were more important to the vaccinated group: “Baby needs one vaccination less”; “receive vaccination through midwife”; “know how to receive MPV”.

Vaccinated pregnant women rated the consideration “Baby would need first vaccination at a later age” of higher importance, and were most frequently of avoidant and impulsive decision-making styles. Vaccinated pregnant women reported greater trust in their midwife, PHI, and PHS, and more often had an older child the unvaccinated pregnant women. Vaccinated pregnant women were more frequently middle-level educated than unvaccinated pregnant women, who were mostly highly educated. Compared to vaccinated non-pregnant women, unvaccinated non-pregnant women more often of Dutch descent.

The most frequently reported reason for not being vaccinated in both pregnant and non-pregnant women was not knowing MPV existed (35% vs 24% respectively). For pregnant women, the next most common reasons were: “I am not yet eligible to receive MPV because my gestational age is less than 28 weeks” (21%) and “MPV was not included in the NIP” (15%). For non-pregnant women the next most common reasons were that they “did not know enough about MPV” (22%), “MPV was not included in the NIP” (19%), and that their “HCW did not advise them to get vaccinated during their earlier pregnancy” (18%).

## Discussion

### Main results

Before the inclusion of the MPV in the Dutch NIP at the end of 2019, when MPV was only available as an out-of-pocket purchase, almost two-thirds of women were aware of the MPV. Awareness was higher among women who were pregnant at the time of survey completion compared to women who were not pregnant but had a child younger than two years (respectively 69% versus 56%). Awareness among pregnant women was likely higher due to being actively informed about the MPV by their midwife. Vaccine uptake was 58% among engaged pregnant women who were eligible for vaccination, and 38% among engaged non-pregnant women. Furthermore, despite different vaccination rates between groups, most women were classified as engaged as they felt that MPV was an important topic to them. As non-pregnant women were less likely to have encountered the new MPV communication materials issued in 2017 and 2018, the lower awareness and vaccine uptake of MPV in this group indicates that the additional communication efforts to improve awareness of MPV seem to have been a success.

The information leaflets, flyers, and posters used for MPV information communication are easily implemented in daily practice. As such, these methods could also be considered for other non-NIP vaccines, such as the Rotavirus vaccine and the human papillomavirus (HPV) vaccine for boys. While providing information regarding additional, non-NIP vaccines is not standard practice in many countries, this may be a feasible method that seems to increase awareness of additional vaccines among the public, as well as among HCWs.

### Awareness of MPV

The significant, overall difference in awareness between pregnant and non-pregnant women could be explained by information provision routes relying on midwives. This information route (through consultation and information flyers) was more accessible to, and more frequently reported by, pregnant women than non-pregnant women. Of non-pregnant women who were aware of MPV, most reported having used the PHI website and traditional media as an information source. These sources were also available to (and used by) pregnant women. Results from previous studies have shown that overall awareness of a topic can be increased by using multiple information delivery routes [15–17]. In this study, most women who were unaware of the MPV indicated needing further information and preferred to be informed by their midwives, either through in-person consultation or by receiving a flyer about MPV. Providing information regarding additional vaccines is not a standard procedure in many countries. However, it is feasible and seems to increase awareness about additional vaccines among public as well as HCWs.

### Engagement with MPV

Most aware women indicated MPV was important to them. These women considered themselves better able to distinguish information and assess the reliability of particular information and its source, and felt that most women would get vaccinated during their pregnancy. These results can be explained by certain cognitive biases; mental short-cuts people make when making decisions. For example, people tend to think that their beliefs and actions are relatively widespread through the general population; known as consensus bias. People also attribute positive terms, such as the ability to distinguish information and assess the reliability of information sources, more often to themselves than others; known as blind-spot bias [18–21].. This might especially be relevant to topics people consider important and they will tend to think other people agree with them [22, 23].

Pregnant women who were aware of, but unengaged with MPV scored lower on trust in all three information sources (e.g. PHI, midwives, and PHS), whilst unengaged non-pregnant women who were aware of the MPV scored lower on trust in PHI and PHS. Other research has shown that trust in information sources is as essential as the actual content of the information [24, 25].

### Information seeking behaviour

Although all women in this stage were engaged, pregnant women may have considered the MPV to be more relevant to them, as most of them still had to decide about MPV soon. This increased situational topic salience for pregnant women may have increased the likelihood of searching for information to support their imminent MPV decision, which was seen in this study. Importantly, even women who reported to be unaware of the MPV indicated that they felt a need for information regarding the MPV. Non-pregnant women mostly reported having used the PHI website to search for information. This underscores that the extra communication efforts information materials (e.g. MPV flyers provided by midwives) may have been less available to non-pregnant women at the time of their previous and most recent pregnancy. The rational decision style was most often reported among women seeking information, which is in line with our expectations and previous research [12]. A rational decision-making style requires processing a lot of information from different sources, contemplating the pros and cons of accepting MPV. This must be done whilst balancing considerations of their beliefs, experiences, and feelings about vaccination, as well as the influence of the social environment [26–28]. However, non-pregnant women who did not seek MPV information identified more as having an avoidant decision-making style. This indicates that irrespective of extra communication efforts, these women may be less likely to seek or require additional MPV information overall.

### Vaccination behaviour

The abundance of available information about MPV for pregnant sample members may have had a positive influence on awareness and vaccine uptake. Relatively more engaged pregnant women reported themselves to have been vaccinated and awareness was higher compared to engaged non-pregnant women. However, other factors, such as educational level, may have played a role. Our sample consisted of relatively few higher-educated pregnant women, and higher levels of education have been previously reported to correlate with an increased hesitancy about vaccination [29–31].

Similar to engaged women, vaccinated women felt that they were more capable of distinguishing information and assessing the reliability of particular sources of information, and felt that most women will get vaccinated during their pregnancy. Among engaged pregnant women, those that were vaccinated reported higher levels of trust in all information sources, and more often already had an older child, when compared to non-vaccinated pregnant women. Having an older child may indicate women were more familiar with childhood vaccinations. As such, pregnant women with older children may have felt less overwhelmed by the influx of information about all aspects of pregnancy they received during their current pregnancy. Vaccinated engaged women also frequently stated that vaccination is “something you just do” and the decision was made without thorough deliberation, corresponding to the predominantly avoidant and impulsive decision-making styles we saw in this group. Comparable results were found in a study on vaccine acceptance among parents in the Netherlands [32]. The reason most reported for not being vaccinated during their (recent) pregnancy was engaged women did not know (enough) about MPV or not (yet) being eligible to receive MPV.

### Practical implications

There are multiple ‘routes’ to increase awareness of vaccinations, such as websites, information flyers, and directly through HCWs. This study emphasizes that it is essential to include and reach all concerned HCWs, as they are a main, and preferred, source of information for the public. In the Netherlands, there is no direct way for the PHI to contact pregnant women. As such, we propose that the best route to further increase awareness of MPV in the Netherlands is through midwives and gynaecologists. They have an important role in providing relevant information to this target group and, according to our results, are the most trusted sources of information. Before they can begin to disseminate this knowledge to pregnant women, HCWs must firstly be engaged and educated on relevant topics, for example through e-learning [15–17]. This route to informing HCWs, and thereby the public, is relatively easily implemented and may also be effective in increasing vaccine uptake. Communication efforts about additional vaccines should therefore include routes to engage and educate HCWs.

It is may also be important to consider the timing of information provided to pregnant women about pregnancy salient topics. Women reported in this survey a feeling of being overwhelmed by the magnitude of information they received during their pregnancy. Various studies have demonstrated that HCWs are most capable of assessing the appropriate moments to offer information to pregnant women [15, 33–36]. However, as most pregnant women in this study searched for information about MPV during their first or second trimester, we suggest that MPV information may well be best presented no later than the beginning of the second trimester.

### Strengths and limitations

To our knowledge, no previous studies have focused on increasing awareness regarding additional, non-NIP vaccines among target groups. The purpose of this study was to investigate the influence of actively informing the public about MPV through their HCWs on MPV awareness and vaccine uptake. We aimed to explore the different stages of the adapted PAPM relating to the decision-making process of pregnant women regarding MPV. Although this study relates to a specific vaccine and its target group, we feel our findings provide information to support future information campaigns of non-NIP vaccines, to maximise not only the spread of awareness and vaccine uptake but also public health.

However, our findings should be interpreted while considering the limitations and biases of this study. Firstly, due to the explorative cross-sectional design of this study, we cannot identify causal relationships. Further, by including non-pregnant women in our study sample, we may have introduced considerable recall biases. Questions in the survey related to the non-pregnant women’s most recent pregnancy, which may have been up to a maximum of 2 years before the study. Our panel and snowballing recruitment method may also have introduced considerable selection biases. This is evidenced by the relatively high vaccine uptake in our sample (58% of eligible pregnant women and 38% of non-pregnant women) compared to the estimated vaccination coverage in the Netherlands (26% until April 2019) [37]. Additionally, there was no available baseline measurement of MPV awareness before the communication efforts took place, and as such, it is not possible to determine the true impact of these efforts on awareness. However, the focus of this study was to describe the (process of) PAPM during pregnancy in light of the availability of information materials. Finally, some women in Stage 3 of the survey reported that they did not know MPV existed. This result seems contradictory, as only women who indicated they were aware of MPV in earlier stages of the questionnaire could enter Stage 3. Perhaps the framing of the questions reminded women of the DTaP vaccine during the survey, indicating our question wording may require further validation.

## Conclusion

As the preferred and most trusted source of information, midwives have an essential role in increasing awareness of MPV. The PHI website is an often-used source of information, but mostly after women had already become aware of MPV and felt a need for more information. Our findings demonstrate that to reach and inform the broadest range of pregnant women, best increase their awareness, and support their decision-making regarding MPV, multiple information routes are essential. Increasing awareness is the first step to improving public engagement with, and eventual acceptance of, a relatively new vaccine. To increase awareness, appropriate healthcare workers should be encouraged to actively inform target groups about available, additional vaccinations.

## Supplementary Information

**Additional file 1 Appendix Table 1**. Cronbach’s alpha.

**Additional file 2.**

## Data Availability

The datasets used and/or analyzed during the current study are available from the corresponding author on reasonable request.

## Abbreviations

dTaP: Diphtheria, tetanus, and acellular Pertussis vaccine
GP: General Practitioner
HCW: Health Care worker
MPV: Maternal Pertussis vaccination
NIP: National Immunisation Program
PaPM: Precaution Adoption Process Model
PHI: Public Health Institute
PHS: Public Health Services
YHW: Youth Health care Worker
