# Supplementary material for: How to increase awareness of additional vaccinations; the case of maternal pertussis vaccination
Source: BMC Public Health. 2021 Jun 29;21:1257. doi: 10.1186/s12889-021-11344-0 (PMC8244209; doi:10.1186/s12889-021-11344-0)
Supplement: Supplementary file 1 — Additional file 1 Appendix Table 1. Cronbach’s alpha. [file 12889_2021_11344_MOESM1_ESM.docx]

**How to increase awareness of additional vaccinations; the case of maternal pertussis vaccination**

*Van Zoonen K.*^1^, Ruijs W.L.M.^1^, De Melker H.E^1^, Bongers, M.E.J..^1^, Mollema, L.^1^*

*^1^Centre for Infectious Disease Control, National Institute for Public Health and the Environment (RIVM), Bilthoven, The Netherlands*

**Corresponding author. Centre for Infectious Disease Control, National Institute for Public Health and the Environment (RIVM), PO Box 1, 3720 BA Bilthoven, The Netherlands. Email-address: Kim.van.zoonen@rivm.nl*

# Appendix 1. Table 1

**Appendix Table 1. Cronbach’s alpha**

| **Scale and Items** | **Cronbach’s alpha (if item deleted)** |
| --- | --- |
| **Attitude: I find MPV** | **0.95** |
| very bad – very good | (0.94) |
| very unimportant – very important | (0.94) |
| very useless – very useful | (0.94) |
| very desirable – very undesirable | (0.94) |
| very unpleasant – very pleasant | (0.95) |
| very harmful – very beneficial | (0.95) |
| very disturbing – very soothing | (0.94) |
| **Intention** | **0.98** |
| I am planning on getting vaccinated against Pertussis during my (next) pregnancy | (0.97) |
| I expect to get vaccinated against Pertussis during my (next) pregnancy | (0.96) |
| It is probable that I will get vaccinated against Pertussis during my (next) pregnancy | (0.98) |
| **Injunctive norm** | **0.66** |
| Loved ones would appreciate it if I got vaccinated against Pertussis during my pregnancy | N/A |
| I think my HCW thinks I should get vaccinated against Pertussis during pregnancy | N/A |
| **Critical thinking** | **0.81** |
| I can distinguish between right and wrong information regarding MPV | (0.83) |
| I critically review information I receive from different sources about MPV | (0.68) |
| When reading information about MPV I take into account the reliability of the source | (0.73) |
| **General Decision Making Style (GDMS)** | |
| **Rationality** | **0.76** |
| I double check my information sources to be sure I have the facts before making a decision | (0.72) |
| I make decisions in a logical and ordered manner | (0.75) |
| To me. making decisions takes considerable thought | (0.71) |
| When making a decision. I think about the different options to reach my goal | (0.72) |
| I research all my options before I make a decision | (0.71) |
| **Intuitive** | **0.82** |
| When I make a decision. I mostly trust my instincts | (0.77) |
| When I make a decision it is more important I feel it is the right decision rather than me having a rational or sensible reason for that decision | (0.82) |
| I trust my inner feelings and responses when I make a decision | (0.75) |
| I trust my instincts when making a decision | (0.76) |
| In general I make decision that feel good to me | (0.80) |
| **Dependent** | **0.76** |
| I hardly ever make important decisions without asking other people’s advice | (0.74) |
| I use other people’s advice when I have to make important decisions | (0.72) |
| When making important decisions I appreciate people pointing me in the right direction | (0.71) |
| I regularly need the help of others when making important decisions | (0.70) |
| If I have the support of others it is easier for me to make important decisions | (0.70) |
| **Avoidant** | **0.91** |
| I postpone making decisions. because thinking about them gives me a feeling of unrest | (0.90) |
| I avoid making important decisions until the pressure is too high | (0.89) |
| If I can I postpone making decisions | (0.88) |
| I procrastinate making important decisions | (0.88) |
| I generally make important decisions at the last minute | (0.89) |
| **Impulsive** | **0.73** |
| I base my decision on what feels right at that moment | (0.75) |
| In general I make quick decisions | (0.67) |
| I usually make impulsive (spontaneous) decisions | (0.64) |
| I often make decisions without thinking about them | (0.68) |
| I take decisions quickly | (0.66) |
