# Supplementary material for: How to increase awareness of additional vaccinations; the case of maternal pertussis vaccination
Source: BMC Public Health. 2021 Jun 29;21:1257. doi: 10.1186/s12889-021-11344-0 (PMC8244209; doi:10.1186/s12889-021-11344-0)
Supplement: Supplementary file 2 — Additional file 2. [file 12889_2021_11344_MOESM2_ESM.docx]

**How to increase awareness of additional vaccinations; the case of maternal pertussis vaccination**

*Van Zoonen K.*^1^, Ruijs W.L.M.^1^, De Melker H.E^1^, Bongers, M.E.J..^1^, Mollema, L.^1^*

*^1^Centre for Infectious Disease Control, National Institute for Public Health and the Environment (RIVM), Bilthoven, The Netherlands*

**Corresponding author. Centre for Infectious Disease Control, National Institute for Public Health and the Environment (RIVM), PO Box 1, 3720 BA Bilthoven, The Netherlands. Email-address: Kim.van.zoonen@rivm.nl*

**Questionnaire maternal pertussis vaccination**

**Welcome!**

The National Health Institute for Public Health and the Environment (RIVM) is conducting research into the maternal pertussis vaccination. You are invited to participate because you are either pregnant or have a child that is younger than 2 years old. With the results of the current study we would like to update and improve our information materials regarding the maternal pertussis vaccination.

The maternal pertussis vaccination will protect newborn baby against pertussis until they are old enough to receive their vaccinations. The maternal pertussis vaccination will also protect you as a mother against pertussis. The vaccination will most likely be included in the national immunization program at the end of 2019. At that moment, pregnant women will receive an invitation. Women today are already able to receive the maternal pertussis vaccination, however this is at your own cost.

**The survey**

In this survey we ask what you may have already heard or read about the maternal pertussis vaccination and how you feel about this vaccination. It consists of 3 parts and will take approximately 20 minutes to finish.

- Part 1 includes questions regarding your information need and information sources which you might have used or prefer to use.
- Part 2 includes questions on what you may already know and how you feel about the maternal pertussis vaccination.
- Part 3 includes more demographic questions, for example about your family, work, etc.

The survey also includes some statements. Some of which might seem identical, however these are necessary to make sure your opinions and thoughts are as clear and complete to us as possible.

**Processing your personal data**

We do not need to know your name and/or address, however we would like to know some other information like your zipcode. This information will be stored for 10 years as is required by law. Only the involved researchers will have access to the information you provide. The RIVM has strict rules and guidelines for taking the utmost professional care regarding your privacy, for more information go to [www.rivm.nl/Privacybeleid](http://www.rivm.nl/Privacybeleid).

You are, of course, free to end or decline your participation without providing us with a reason to do so. However, we hope you are willing to complete the entire survey, because your answers matter to us.

**Staying informed about the research**

If you would like to be informed about the results of this research, you can indicate so at the end of the survey. If you have any questions or remarks regarding your participation or the survey, please feel free to contact the researchers using this email-address:

[onderzoekvaccinatie@rivm.nl](mailto:onderzoekvaccinatie@rivm.nl)

or call us at 030-2742484 (Kim van Zoonen).

Good luck filling out the survey!

Kind regards,

Kim van Zoonen

Part 1.

The first few questions focus on what you may have already heard/read about the maternal pertussis vaccination and possible information seeking behavior.

Even if you have not yet heard/read about maternal pertussis vaccination and have not searched for any information regarding this vaccination, we are interested in your opinion.

1. Have you heard/read about maternal pertussis vaccination during your (most recent) pregnancy?
   - 1. Yes
     2. No
2. What was your information source? [If Q1 = Yes]

*You are able to choose more than one source.*

1. Midwife
2. GP
3. Municipal Health Services
4. Gynaecologisch
5. (employee at the) Infant welfare centre
6. RIVM
7. Government
8. Other pregnant women
9. Other, namely;_________________________________
10. How did you hear/read the information? [If Q1 = Yes]

*You are able to choose more than one answer.*

1. In a consultation with a health care professional
2. Through a flyer specifically focused on maternal pertussis vaccination
3. Through the website of the RIVM
4. Through the internet
5. Through social media (e.g. Facebook, Instagram or Twitter)
6. Through other media (e.g. television, newspaper of magazine)
7. Through group sessions with other pregnant women (e.g. ‘centering pregnancy’ meetings)
8. Other, namely;_______________________
9. Would you have like to have heard/read the information? [If Q1 = No]
   - 1. Yes
     2. No
10. Whom would you prefer to have provided you the information? [If Q1 or Q4 = Yes]
    - 1. Midwife
      2. GP
      3. Municipal Health Services
      4. Gynaecologisch
      5. (employee at the) Infant welfare centre
      6. RIVM
      7. Government
      8. Other pregnant women
      9. Other, namely;_________________________________
11. How would you prefer to receive/have received the information? [If Q1 or Q4 = Yes]
12. In a consultation with a health care professional
13. Through a flyer specifically focused on maternal pertussis vaccination
14. Information added to the existing flyer ‘pregnant!’
15. Through the website of the RIVM
16. Through social media (e.g. Facebook, Instagram of Twitter)
17. Through other media (e.g. television, newspaper of magazine)
18. Through e-mail
19. Through a large scale information campaign by the government
20. Through a mobile app
21. Through group sessions with other pregnant women (e.g. ‘centering pregnancy’ meetings)
22. With an online decision aid-tool (i.e. a program designed to inform and help you in making a decision regarding maternal pertussis vaccination)
23. Other, namely;_______________________
24. What is the most important reason you prefer to not receive any information? [If Q4 = No]

_____________________________________________

1. Did you (actively) search for (more) information regarding the maternal pertussis vaccination during your (most recent) pregnancy?
2. Yes
3. No
4. When did you seek this information? [If Q8 = Yes]
5. Before my (most recent) pregnancy
6. First trimester (between 0 en 12 weeks pregnant)
7. Second trimester (between 13 en 26 weeks pregnant)
8. Third trimester (between 27 en 42 weeks pregnant)
9. After my (most recent) pregnancy
10. Where did you find the (extra) information? [If Q9 = Yes]

*You can choose more than 1 answer.*

1. At my GP
2. At my midwife
3. At the infant welfare centre
4. At the municipal health care centre
5. On the website of the RIVM
6. On another website
7. In scientific literature
8. On the television
9. In a newspaper/magazine
10. Other pregnant women
11. On social media (e.g. Facebook, Instagram of Twitter)
12. Friends/family
13. Other, namely:_________________
14. On which website did you find the (extra) information? [If Q10 = vi]

_________________________________________________________

1. In which newspaper and/or magazine did you find the (extra) information? [If Q10 = ix]

________________________________________________________

Below you find a statement regarding the information you have searched.

Could you please indicate how reliable you felt the information was.

*[1 = very unreliable – 7 = very reliable]*

1. How reliable was the information regarding […]?[for every indicated source in Q10]

Below you will find several statements. Could you please indicate to what extent you experience trust.

*[1 = absolutely no trust – 7 = a lot of trust]*

1. How trustworthy do you find the information regarding maternal pertussis vaccination you receive(d) from you midwife and/or gynecologist?
2. How trustworthy do you find the information regarding maternal pertussis vaccination you receive(d) from the RIVM?
3. How trustworthy do you find the information regarding maternal pertussis vaccination you receive(d) from the infant welfare centre?

Could you please indicate to what extent you agree or disagree with the statements below.

*[1 = totally disagree – 7 = totally agree]*

1. I am able to discriminate between correct en incorrect information regarding maternal pertussis vaccination
2. I examine information regarding maternal pertussis vaccination that I receive from multiple sources critically/thoroughly.
3. I take the credibility of a source into account when I process information regarding maternal pertussis vaccination.
4. The maternal pertussis vaccination is an important topic to me.

Part 2.

In this part we ask questions regarding your knowledge and feelings about maternal pertussis vaccination. Even if you do not know the answer we ask you to choose the one you think is best.

*1 = Very little – 5 = A lot*

1. How much do you think you know about maternal pertussis vaccination?
2. Do you feel you know enough to make a decision regarding maternal pertussis vaccination?
3. Yes
4. No
5. What sources did you use to boost your knowledge?

*You can choose more than one option.*

1. Health care professional
2. At work/my colleagues
3. The Internet
4. Website RIVM
5. Social media (e.g. Facebook, Instagram of Twitter)
6. Family
7. Friends
8. Other, namely:____________________________________
9. How many hospitalizations of newborn babies due to pertussis can be prevented by vaccination during pregnancy?
10. Approximately 40%
11. Approximately 70%
12. Approximately 90%
13. How many newborn babies are admitted to the hospital every year due to pertussis?
14. Approximately 10
15. Approximately 100
16. Approximately 500
17. Pertussis in newborn babies does not have severe course.
    - 1. True
      2. False
      3. I don’t know
18. Maternal pertussis vaccination has adverse effects on the unborn child.
19. True
20. False
21. I don’t know
22. I find the maternal pertussis vaccination:
23. 1= very bad – 7 = very good
24. 1= very unimportant – 7 = very important
25. 1= very unnecessary – 7= very necessary
26. 1= Undesirable – 7= Desirable
27. 1= very uncomfortable – 7= very comfortable
28. 1= very harmful – 7= very unharmful
29. 1= very disturbing – 7= very reassuring
30. Whom would you consult whether or not to receive the maternal pertussis vaccination?

*You can choose more than one option.*

1. With my partner
2. With my familymember(s)
3. With my friends
4. With other pregnant women
5. With colleagues
6. With my midwife
7. With an employee from the infant welfare centre/youth health care worker
8. With my GP
9. With my gyneacologist
10. Other; namely______________________

Below you find some statement. Could you please indicate to what extent you agree or disagree with each statement.

*[1 = totally disagree – 7 = totally agree]*

1. Most pregnant women will choose to receive the maternal pertussis vaccination.
2. My loved ones will appreciate it when I receive the maternal pertussis vaccination.
3. I think my midwife feels I should get the maternal pertussis vaccination.

Could you please indicate to what extent the next statements apply to you.

*[1=Absolutely not – 7=Absolutely]*

1. I intend to get vaccinated against pertussis during my (next) pregnancy.
2. I expect to get vaccinated against pertussis during my (next) pregnancy.
3. It is probable that I will get vaccinated against pertussis during my (next) pregnancy.
4. Are you planning to get your (future) children vaccinated?
5. Yes, completely following the NIP
6. Yes, but not completely according tot he NIP
7. No
8. I don’t know (yet)

COuld you please indicate to what extent you agree with the next statements.

*1= totally disagree – 7 = totally agree*

1. If my newborn baby would need less vaccinations against pertussis due to the maternal pertussis vaccination that would be a reason to get the maternal pertussis vaccination.
2. If the maternal pertussis vaccination would be administered by my midwife it would be a reason to get the vaccination.
3. If I would want to get the maternal pertussis vaccination I would know what to do.
4. If the maternal pertussis vaccination is included in the NIP (and thus free of charge) it would be a reason to get it.
5. If my newborn would receive its first vaccinations at a later age due to the maternal pertussis vaccination that would be a reason to get the maternal pertussis vaccination.
6. If the maternal pertussis vaccination would be administered by someone else than my GP or midwife it would be a reason to not receive the vaccination.

Below you will find some statement about how people make important decision. There are no right or wrong answers. We would like you to take a moment and think about how you usually make decisions. Therefore, it is important you answer the question regarding your actual behavior.

*[1=strongly disagree, 2=disagree, 3=neutral (neither agree nor disagree), 4=agree, 5=strongly agree]*

1. When I make decisions, I tend to rely on my intuition.
2. I rarely make important decisions without consulting other people
3. When I make a decision, it is more important for me to feel the decision is right than to have a rational reason for it
4. I double check my information sources to be sure I have the right facts before making decisions
5. I use the advice of other people in making my important decisions
6. I put off making decisions because thinking about them makes me uneasy
7. I make decisions in a logical and systematic way
8. When making decisions I do what feels natural at the moment
9. I generally make snap decisions
10. I like to have someone steer me in the right direction when I am faced with important decisions
11. My decision-making requires careful thought
12. When making a decision, I trust my inner feelings and reactions
13. When making a decision, I consider various options in terms of a specified goal
14. I avoid making important decisions until the pressure is on
15. I often make impulsive decisions
16. When making decisions, I rely upon my instincts
17. I generally make decisions that feel right to me
18. I often need the assistance of other people when making important decisions
19. I postpone decision making whenever possible
20. I often make decisions on the spur of the moment
21. I often put off making important decisions
22. If I have the support of others, it is easier for me to make important decisions
23. I generally make important decisions at the last minute
24. I make quick decisions
25. I explore all of my options before making a decision

Part 3.

Finally, we would like the ask you some questions about you and your family.

1. What is your zipcode (1111)?
2. What is your age in years?
3. What is your country of birth?
   - 1. Netherlands
     2. Morocco
     3. Turkey
     4. Surinam
     5. Dutch Antilles
     6. Other, Namely:____________________
4. What is your highest completed education level?
5. No education (Did not finish primary school)
6. Primary education (primary school, specialized primary school)
7. Prevocational education (e.g. LTS, LEAO, LHNO, LBO, VMBO-(BB, KB, GL))
8. Middle general continued education (e.g. MAVO, (M)ULO, MBO-kort, VMBO-TL)
9. Secondary vocational education and vocational training (e.g. MBO-lang, MTS, MEAO, BOL, BBL, INAS)
10. Higher general continued education and primary education (e.g. HAVO, VWO, Atheneum, Gymnasium, HBS, MMS)
11. Higher education (e.g. HBO, HTS, HEAO)
12. Scientific education (University)
13. Other, namely; ____________________________________
14. Are you employed as a health care professional?
15. Yes
16. No
17. Do you have children?
18. Yes
19. No
20. What is the age of your youngest child [If Q74 =ja]

___ Year __ month(S)


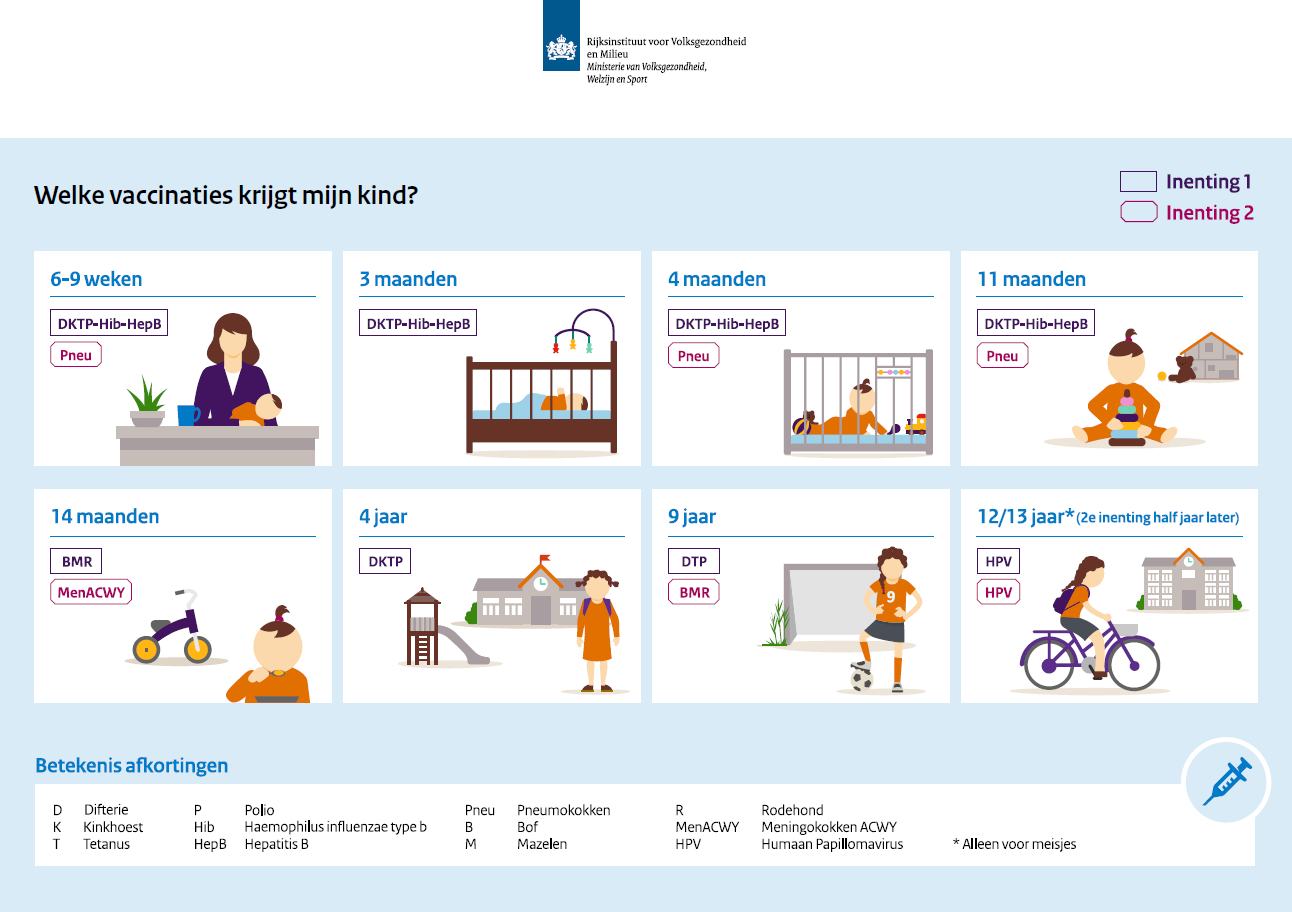


1. Does your youngest child participate in the national immunization program (NIP)? [If Q74 =yes]
   - 1. Yes, completely (received all vaccinations recommended for his/her age)
     2. Yes, partially (he/she has not received all vaccinations recommended for his/her age)
     3. No
     4. I do not know
2. Are you currently pregnant?
3. Yes
4. No
5. How many weeks are you pregnant? [If Q77 = Yes)
6. The checkups during my (last) pregnancy was/is done by:
7. Midwife
8. Hospital
9. Other, namely;____________________________________
10. Did you receive the maternal pertussis vaccination during your (last) pregnancy?
    - 1. Yes
      2. No
11. Who provided the maternal pertussis vaccination? [If Q80 = Yes]
12. GP
13. Midwife
14. Municipal health service
15. Other, Namely:______________________
16. What is the most important reason to not receive the maternal pertussis vaccination? [If Q80 = No]
17. I was/am unaware of the existence of the maternal pertussis vaccination
18. I have objections against vaccinations during pregnancy.
19. I don’t know enough about the maternal pertussis vaccination to get vaccinated.
20. I cannot afford it.
21. The maternal pertussis vaccination is not included in the NIP
22. The maternal pertussis vaccination is not advised by my health care professional.
23. Other, namely:___________________
24. Were you vaccinated according to the NIP as a child?
25. Yes, I received all vaccinations
26. Yes, however I did not receive all vaccinations
27. No
28. I don’t know
29. How did you receive our survey?
    - 1. Friends
      2. Family
      3. Colleagues/work
      4. Other pregnant women
      5. Through Panelcompany
      6. Website, namely:________________
30. Finally, we would like to hear more from you during a short interview. During this interview we would like to offer you the possibility to elaborate about your wishes, opinion and questions regarding the maternal pertussis vaccination. Would you be willing to participate in such an interview and might we contact you?
31. Yes, [email address]
32. No
33. [If Q85 = Yes] I give permission to save my email address in order for the RIVM to contact me fora n interview regarding the maternal pertussis vaccination.
34. I would like to be kept up to date regarding results from this study?
    - 1. Yes, [email address]
      2. No, thank you.
35. Do you have any remarks, questions or anything else which you would like us to know?

_________________________________________________________________________

You have reached the end of the survey and we would like to thank you very much for your participation in our study.
